# Incidence and Outcomes of Non–Ventilator-Associated Hospital-Acquired Pneumonia in 284 US Hospitals Using Electronic Surveillance Criteria

**DOI:** 10.1001/jamanetworkopen.2023.14185

**Published:** 2023-05-18

**Authors:** Barbara E. Jones, Aaron L. Sarvet, Jian Ying, Robert Jin, McKenna R. Nevers, Sarah E. Stern, Aileen Ocho, Caroline McKenna, Laura E. McLean, Matthew A. Christensen, Russell E. Poland, Jeffrey S. Guy, Kenneth E. Sands, Chanu Rhee, Jessica G. Young, Michael Klompas

**Affiliations:** 1 Division of Pulmonary and Critical Care Medicine, University of Utah, Salt Lake City; 2 VA Salt Lake City Health Care System, Salt Lake City, Utah; 3 Department of Population Medicine, Harvard Medical School and Harvard Pilgrim Health Care Institute, Boston, Massachusetts; 4 Division of Epidemiology, University of Utah, Salt Lake City; 5 HCA Healthcare Inc, Nashville, Tennessee; 6 Division of Allergy, Pulmonary, and Critical Care Medicine, Vanderbilt University Medical Center, Nashville, Tennessee; 7 Department of Medicine, Brigham Women’s Hospital, Boston, Massachusetts

## Abstract

**Importance:**

Non–ventilator-associated hospital-acquired pneumonia (NV-HAP) is a common and deadly hospital-acquired infection. However, inconsistent surveillance methods and unclear estimates of attributable mortality challenge prevention.

**Objective:**

To estimate the incidence, variability, outcomes, and population attributable mortality of NV-HAP.

**Design, Setting, and Participants:**

This cohort study retrospectively applied clinical surveillance criteria for NV-HAP to electronic health record data from 284 US hospitals. Adult patients admitted to the Veterans Health Administration hospital from 2015 to 2020 and HCA Healthcare hospitals from 2018 to 2020 were included. The medical records of 250 patients who met the surveillance criteria were reviewed for accuracy.

**Exposures:**

NV-HAP, defined as sustained deterioration in oxygenation for 2 or more days in a patient who was not ventilated concurrent with abnormal temperature or white blood cell count, performance of chest imaging, and 3 or more days of new antibiotics.

**Main Outcomes and Measures:**

NV-HAP incidence, length-of-stay, and crude inpatient mortality. Attributable inpatient mortality by 60 days follow-up was estimated using inverse probability weighting, accounting for both baseline and time-varying confounding.

**Results:**

Among 6 022 185 hospitalizations (median [IQR] age, 66 [54-75] years; 1 829 475 [26.1%] female), there were 32 797 NV-HAP events (0.55 per 100 admissions [95% CI, 0.54-0.55] per 100 admissions and 0.96 per 1000 patient-days [95% CI, 0.95-0.97] per 1000 patient-days). Patients with NV-HAP had multiple comorbidities (median [IQR], 6 [4-7]), including congestive heart failure (9680 [29.5%]), neurologic conditions (8255 [25.2%]), chronic lung disease (6439 [19.6%]), and cancer (5,467 [16.7%]); 24 568 cases (74.9%) occurred outside intensive care units. Crude inpatient mortality was 22.4% (7361 of 32 797) for NV-HAP vs 1.9% (115 530 of 6 022 185) for all hospitalizations; 12 449 (8.0%) were discharged to hospice. Median [IQR] length-of-stay was 16 (11-26) days vs 4 (3-6) days. On medical record review, pneumonia was confirmed by reviewers or bedside clinicians in 202 of 250 patients (81%). It was estimated that NV-HAP accounted for 7.3% (95% CI, 7.1%-7.5%) of all hospital deaths (total hospital population inpatient death risk of 1.87% with NV-HAP events included vs 1.73% with NV-HAP events excluded; risk ratio, 0.927; 95% CI, 0.925-0.929).

**Conclusions and Relevance:**

In this cohort study, NV-HAP, which was defined using electronic surveillance criteria, was present in approximately 1 in 200 hospitalizations, of whom 1 in 5 died in the hospital. NV-HAP may account for up to 7% of all hospital deaths. These findings underscore the need to systematically monitor NV-HAP, define best practices for prevention, and track their impact.

## Key Points

**Question:**

What is the incidence and mortality burden of non–ventilator-associated hospital-acquired pneumonia (NV-HAP) in US hospitals?

**Findings:**

In a retrospective cohort analysis of 284 hospitals using an electronic surveillance definition applied to detailed clinical data, there was an estimated 0.55 NV-HAP events per 100 hospitalizations, inpatient mortality of 22.4%, and an additional 8.0% discharged to hospice. NV-HAP may account for an estimated 7.3% of all hospital deaths.

**Meaning:**

These findings suggest that NV-HAP is a common and deadly complication of hospitalization that could account for up to 1 in 14 hospital deaths.

## Teaser

This cohort study examines the incidence, variability, outcomes, and population attributable mortality of non–ventilator-associated hospital-acquired pneumonia in the US.

## Introduction

Hospital-acquired pneumonia is the most common health care-associated infection in the United States and is associated with high morbidity, mortality, and health care use.^1,2^ Most cases occur in nonventilated patients. Crude mortality rates for non–ventilator-associated hospital-acquired pneumonia (NV-HAP) are similar to those for ventilator-associated pneumonia (VAP).^1,3,4^ However, most hospitals only have surveillance and prevention programs for VAP but not for NV-HAP.

Hospitals’ limited attention to NV-HAP is partly due to difficulty defining and tracking NV-HAP and a limited understanding of its burden and preventability. The surveillance definitions published by the US Centers for Disease Control and Prevention National Healthcare Safety Network (CDC-NHSN)^5^ include many subjective and ambiguous criteria that are complicated and difficult to apply in a clear and consistent manner, even for experienced clinicians.^6,7^ These include changes in oxygenation, the quality and quantity of respiratory secretions, and interpreting chest radiographs. These criteria are prone to high levels of human error and interobserver variability and correspond inconsistently with histological pneumonia.^8,9^ Surveillance using discharge diagnosis codes is similarly limited because of their poor sensitivity, specificity, and variability within and between hospitals in how and when codes are applied.^10,11^ These challenges deter hospitals from developing NV-HAP surveillance and prevention programs, complicate the assessment of NV-HAP prevention initiatives, and make it difficult to estimate the national burden of NV-HAP.

Electronic health record (EHR) systems allow for the possibility of identifying NV-HAP using detailed electronic clinical data that can be applied in a consistent, automatable, and efficient manner across hospitals instead of existing approaches based on manual medical record reviews or administrative coding.^12^ This approach does not overcome the limited accuracy of traditional surveillance definitions but does have the potential to generate comparable estimates of incidence and outcomes between hospitals and across time in an efficient and reproducible manner.

We implemented a previously developed and validated electronic NV-HAP surveillance definition^12,13^ predicated upon identifying patients with new and sustained deteriorations in oxygenation, abnormal temperature or white blood cell count, chest imaging, and new antibiotic starts. The electronic surveillance definition was applied to EHR data from a large and diverse set of US hospitals to estimate the incidence and variability of NV-HAP and its association with outcomes. We then used detailed daily clinical data to estimate the attributable population mortality of NV-HAP, accounting for both baseline confounding and time-dependent bias.

## Methods

This retrospective cohort study was approved by the institutional review boards of the Veterans Affairs (VA), University of Utah, and Harvard Pilgrim Health Care Institute. Informed consent was waived because consent would not be feasible and the study posed minimal patient risk. This study followed the Strengthening the Reporting of Observational Studies in Epidemiology (STROBE) reporting guideline.

### Design, Data Source, and Study Populations

The electronic NV-HAP surveillance definition was retrospectively applied to EHR data drawn from 284 acute care hospitals in the Veterans Affairs (VA) health care system and HCA Healthcare networks. The VA network is the largest integrated health care network in the US and includes hospitals in all 50 US states. All VA hospitals use the same clinical EHR, Veterans Health Information Systems and Technology Architecture (VistA). Data were accessed from VistA through the Veterans Informatics and Computing Infrastructure, a computing environment that stores clinical data for research purposes.^14^ HCA Healthcare is the largest private hospital network in the US and includes large, medium, and small hospitals in 21 US states. The majority of HCA Healthcare hospitals use a common EHR with data that are aggregated and validated centrally. HCA’s centralized data has previously been used to support large-scale surveillance studies in multiple domains, including sepsis, opioid use, and health care-associated infections.^15,16,17^

We identified all hospitalizations for adults ages 18 years or older admitted to VA acute care facilities between January 1, 2015, and November 30, 2020, and HCA Healthcare facilities between October 1, 2018, and May 31, 2020. For descriptive analyses, we included all hospitalizations. For the weighted analysis estimating attributable mortality risk, we only included hospitalizations of at least 3 days with complete data for the first or second day of admission for the following time-varying variables: hospital service, white blood cell count, hematocrit, platelets, sodium, glucose, and creatinine.

### Electronic NV-HAP Surveillance Definition

The electronic NV-HAP surveillance definition was designed to mirror traditional surveillance definitions.^12^ The definition requires a decrease in oxygen saturation or increase in supplemental oxygen sustained for 2 or more days after 2 or more days of stable or improving oxygenation, plus an abnormal temperature (≤36 °C or ≥38 °C) or white blood cell count (<4000 or ≥12 000 cells/mm^3^), plus completion of chest imaging (x-ray or computed tomography), plus administration of 3 or more days of new antimicrobials starting on the first or secondary day of oxygen deterioration.^12^ Previous studies suggest this definition performs similarly to traditional CDC-NHSN criteria in terms of incidence, mortality, and clinical correlates.^12,18^ Further details regarding these criteria and the SAS code to apply the criteria are available in GitHub and eAppendix 1 in Supplement 1.^19^

We extracted patients’ demographics, comorbidities, vital signs, supplementary oxygen devices, laboratory tests, and discharge diagnosis codes. We defined comorbid conditions (Table 1) using the Elixhauser method applied to the *International Classification of Diseases, Ninth Revision, Clinical Modification *(*ICD-9-CM*)^20^ and the *International Statistical Classification of Diseases, Tenth Revision, Clinical Modification *(*ICD-10-CM*)^21^ codes via software developed by the Agency for Healthcare Research and Quality.^22,23^ We calculated a summary Elixhauser index score using the method of van Walraven.^24^ We extracted the first, minimum, maximum, mean, and median vital signs, pulse oximetry, supplemental oxygen use, and laboratory results for each calendar day of hospitalization. We identified daily specialty service and ward type (medical/surgical ward or intensive care unit). We also extracted facility characteristics, including bed size, geographic region, and teaching status, defined as the presence of graduate medical residents or medical students.

**Table 1.** Characteristics of All Hospitalized Patients and Patients With NV-HAP^a^

Characteristics	Hospitals, No, (%), (N = 284)	Patients, No. (%)		
		All hospitalizations (n = 6 022 185)	NV-HAP (n = 32 797)	NV-HAP per 100 hospitalizations
Hospital				0.55
No. of beds				
1-99	105 (37)	1 011 955 (16.8)	3421 (10.3)	0.34
100-199	82 (29)	2 114 839 (35.1)	10 049 (30.3)	0.48
200-299	46 (16)	1 221 756 (20.3)	7692 (23.5)	0.64
≥300	51 (18)	1 673 635 (27.8)	11 635 (35.8)	0.71
Teaching status				
Teaching	199 (70)	4 526 892 (75.2)	24 657 (75.0)	0.55
Nonteaching	85 (30)	1 495 293 (24.8)	8140 (25.0)	0.56
Region				
Midwest	47 (16)	900 103 (14.9)	3681 (11.2)	0.41
Northeast	30 (11)	469 681 (7.8)	2315 (7.0)	0.49
South	158 (56)	3 597 142 (59.7)	20 817 (63.7)	0.59
West	49 (17)	1 055 259 (17.5)	5984 (18.2)	0.57
Patient				
Age, median (IQR), years	NA	66 (54-75)	69 (61-77)	
Sex				
Female	NA	1 829 475 (30.4)	8556 (26.1)	0.47
Male	NA	4 171 527 (69.3)	24 241 (73.9)	0.58
Race				
Asian	NA	83 374 (1.4)	556 (1.7)	0.67
Black	NA	1 121 432 (18.6)	5325 (16.2)	0.47
White	NA	4 238 887 (70.4)	23 683 (72.2)	0.56
Other or missing^b^	NA	554 762 (9.2)	3084 (9.4)	0.56
Hospital service				
Cardiology	NA	186 005 (3.1)	2252 (6.9)	1.21
Medicine	NA	3 496 131 (58.1)	19 959 (60.9)	0.57
Surgery	NA	1 631 261 (27.1)	9388 (28.6)	0.58
Neuroscience	NA	53 383 (0.9)	483 (1.5)	0.90
Oncology	NA	32 407 (0.5)	468 (1.4)	1.44
Other	NA	622 998 (10.3)	247 (0.8)	0.04
Comorbidities				
Congestive heart failure	NA	972 091 (16.1)	9680 (29.5)	1.00
Chronic lung disease	NA	1 018 405 (16.9)	6439 (19.6)	0.63
Diabetes	NA	1 964 829 (32.6)	13 996 (42.7)	0.71
Chronic liver disease	NA	344 240 (5.7)	3437 (10.5)	1.00
Cancer	NA	558 021 (9.3)	5467 (16.7)	0.98
Neurological disease	NA	808 271 (13.4)	8255 (25.2)	1.02
Chronic kidney disease	NA	1 087 481 (18.1)	10 674 (32.5)	0.98
No comorbidities	NA	694 357 (11.5)	323 (1.0)	0.05
Median No. of comorbidities (IQR)	NA	3 (2-5)	6 (4-7)	NA
Median Elixhauser Index	NA	3 (0-9)	14 (7-21)	NA
Outcomes				
Median hospital length-of-stay (IQR)	NA	4 (3-6)	17 (11-26)	NA
Discharge disposition				
Home	NA	4 851 605 (80.6)	12 449 (38.0)	NA
Rehabilitation facility	NA	124 751 (2.1)	1565 (4.8)	NA
Skilled nursing facility	NA	521 093 (8.7)	5783 (17.6)	
Hospice	NA	84 676 (1.4)	2629 (8.0)	NA
Death	NA	115 530 (1.9)	7361 (22.4)	NA

Abbreviations: NA, not applicable; NV-HAP, non–ventilator-associated hospital-acquired pneumonia.

^a^ NV-HAP per 100 hospitalizations for different strata are also shown. Frequencies are reported as No. (%). Continuous values are reported as median (IQR).

^b^ Categories included in the other classification are as follows—for HCA: American Indian or Alaskan Native, Hawaiian or Pacific Islander, Hispanic, Multiracial, other, unknown; for VA: American Indian or Alaska Native, Native Hawaiian or other Pacific Islander, mixed, or missing.

Race data were collected according to the data entered into the EHR. The majority of facilities collect race as self-reported. Race was used as a time-fixed covariate since it is a marker for additional baseline risk. Ethnicity was not collected because it was not consistently available. Categories of race included were Asian, Black, White, and other. For HCA, the other category included American Indian or Alaskan Native, Hawaiian or Pacific Islander, Hispanic, Multiracial, other, unknown, and for VA, other included American Indian or Alaska Native, Native Hawaiian or other Pacific Islander, mixed, or missing. This is described in Table 1.

### Medical Record Review

The medical records of 250 randomly selected VA hospitalizations meeting electronic NV-HAP criteria were reviewed by 3 physicians (B.E.J., S.E.S., M.A.C.). Each record was independently reviewed by 2 physicians to determine: (1) whether the bedside clinical team documented NV-HAP; (2) whether the event met CDC-NHSN PNU criteria for NV-HAP^5^; (3) whether NV-HAP was recorded in the discharge summary; and (4) whether the reviewer judged the case to be consistent with NV-HAP. Details, including the medical record review guide with specific definitions, are available in eAppendix 2 in Supplement 1 and separate work.^13^

### Statistical Analysis

#### Descriptive Analysis

Incidence of NV-HAP (events per 100 hospitalizations and 1000 patient-days), patient characteristics, and inpatient mortality were calculated. To visualize facility-level variation, we plotted the incidence of NV-HAP surveillance events per 100 hospitalizations for each facility, ranked by incidence. Among the cases undergoing medical record review, we calculated the positive predictive value of electronic NV-HAP surveillance criteria vs bedside clinicians, CDC/NHSN criteria, and reviewers and assessed interrater agreement using Cohen κ.

#### Estimating the Attributable Mortality of NV-HAP

We estimated the attributable mortality of NV-HAP by modeling what could happen to population mortality if we could eliminate NV-HAP from the population theoretically. This is not to say that we believe that NV-HAP can be entirely eliminated; rather, this is a statistical approach for estimating attributable mortality that effectively handles time-dependent bias.^25^ This approach can account for complex time-varying confounding that cannot adequately be addressed using standard regression procedures and that risk spurious associations.^26^ We modeled the cumulative risk of the primary outcome (inpatient death by 60 days follow-up) vs alive discharge (a competing risk for in-hospital mortality) for the entire hospital population with NV-HAP cases included (current care). We then modeled cumulative incidences of inpatient death by 60 days under the theoretical condition of eliminating all NV-HAP cases from the hospital population. We compared these values to estimate the population attributable risk of inpatient death that is associated with NV-HAP.

We used inverse probability weighting^27^ based on an estimated propensity score on each day to adjust for the following time-fixed confounders: hospital characteristics (size, teaching status, and region), patients’ demographics (age, race, or sex), comorbidities, and hospitalization in the preceding 90 days. We also adjusted for the following time-varying confounders, corresponding to the most recent measured value 2 days prior: hospital service, intensive care unit (ICU) status, and markers of illness severity, including oxygen delivery device, pulse oximetry, routine laboratory test results (hematocrit, platelets, sodium, glucose, creatinine), and days since any current routine lab measure. We also adjusted for categories of nonroutine laboratory measures (alanine aminotransferase, total bilirubin, and albumin) as detailed in eAppendix 3 in Supplement 1. The statistical code is available via GitHub.^19^ Missing pulse oximetry or oxygen supplementation were treated as normal.

We used a weighted Aalen-Johansen estimator of the cumulative incidence of inpatient death and discharged alive under (1) current care and (2) hypothetical elimination of NV-HAP.^28^ Daily propensity scores for an NV-HAP event were used to construct weighted time-varying estimates of the hazards for each competing event, which were then used to calculate hazards for each outcome.^27^ Statistical methods are described in full in eAppendix 3 in Supplement 1. Risk ratios and risk differences were calculated at 60 days follow-up by taking the ratio and difference, respectively, of the risk estimate with hypothetical elimination of NV-HAP vs current care. We generated 95% CIs using a nonparametric bootstrap to resample hospitalizations with replacement 500 times.

We conducted exploratory analyses stratified by age (ie, ≤65 years or >65 years), service group (medical, surgical, cardiology, neuroscience, oncology, and other), ICU status on hospital day 3, Elixhauser comorbidity index quartile, hospital number of beds, region, and hospital teaching status. We also conducted a sensitivity analysis using inpatient death vs alive discharge by 30 days of follow-up as a secondary outcome. Hospital-level point estimates and bootstraps were computed separately for VA and HCA in order to maintain data security and then combined using weighted averages, with site-specific weights proportional to sample size (ie, the number of unique hospitalizations associated with each data source). All statistical analyses were performed using SAS version 9.4 (SAS Institute) and RStudio version 1.4 (RStudio).^29^

## Results

### Description of Incidence, Clinical Characteristics, and Outcomes

**Figure 1.**
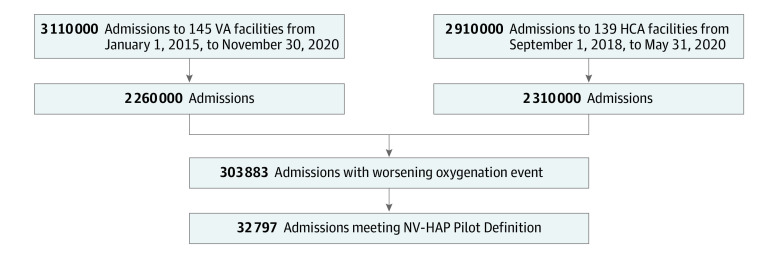
Study Population HCA indicates HCA Healthcare hospitals; NV-HAP, non–ventilator-associated hospital-acquired pneumonia; VA, Veterans Affairs.

**Figure 2.**
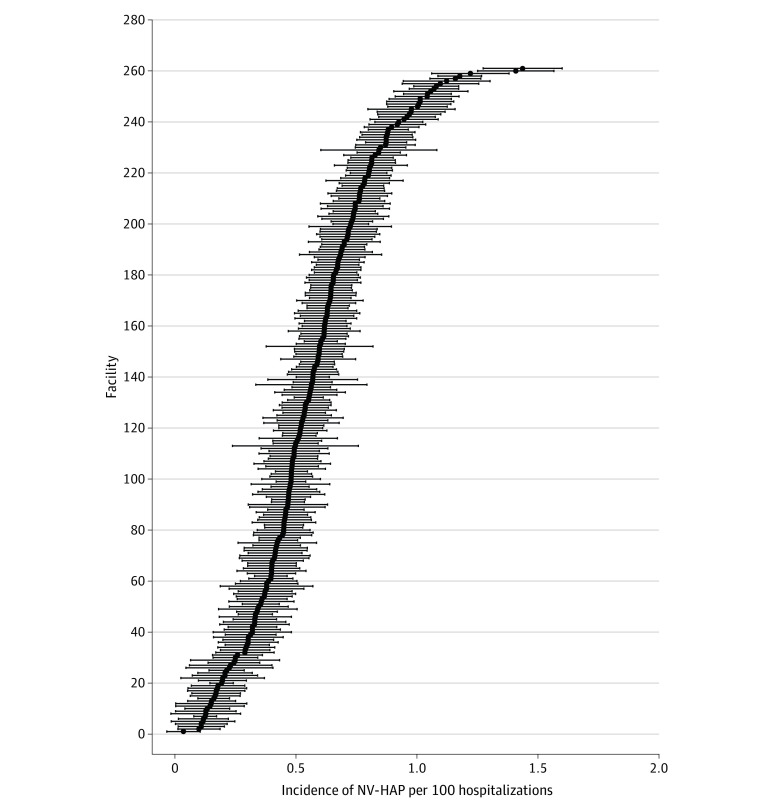
Incidence of NV-HAP Events per 100 Hospital Admissions for Each Facility Caterpillar plot depicting incidence of NV-HAP events per 100 hospital admissions (black marker) and 95% CIs (error bars) for each facility in the study population. NV-HAP indicates non–ventilator-associated hospital-acquired pneumonia.

There were 6 022 185 hospital admissions and 34 110 135 hospital days among all 284 facilities (145 VA hospitals and 139 HCA hospitals) during the study period. Of these, 303 883 admissions met the criteria for sustained deterioration in oxygenation after 2 or more days of stable or improving oxygenation (5.1 [95% CI, 5.03-5.06] per 100 admissions, 8.9 [95% CI, 8.88-8.94] per 1000 hospital days), and 32 797 met surveillance criteria for NV-HAP, for an incidence of 0.54 (95% CI, 0.54-0.55) per 100 admissions and 0.96 (95% CI, 0.95-0.97) per 1000 patient-days (Figure 1). At the facility level, incidence rates ranged from 0 to 1.44 events per 100 admissions (median [IQR], 0.52 [0.35-0.69]) (Figure 2). Patients’ characteristics are shown in Table 1, and clinical characteristics of NV-HAP events are shown in Table 2. Patients with NV-HAP were older (median [IQR] age, 69 [61-77] vs 66 [54-75] years among all hospitalized patients) and most had multiple comorbidities (median [IQR], 6 [4-7]), most commonly congestive heart failure (9680 [29.5%]), neurologic disease (8255 [25.2%]), chronic lung disease (6439 [19.6%]), and cancer (5467 [16.7%]). Most cases (24 568 [74.9%]) occurred outside intensive care units. The inpatient mortality rate was 22.4% (7361 of 32 797) among admissions meeting the NV-HAP surveillance definition vs 1.9% (115 530 of 6 022 185) among all other admissions. An additional 2629 of 32 797 of patients (8.0%) with NV-HAP were discharged to hospice vs 84 676 of 6 022 185 (1.4%) among all hospitalizations; only 12 449 (38.0%) of NV-HAP admissions culminated in discharge to home vs 4 851 605 of 6 022 185 (80.6%) among other admissions. Median (IQR) length-of-stay for patients with NV-HAP was 17 (11-26) days vs 4 (3-6) days for the general hospital population.

**Table 2.** Clinical Characteristics and Outcomes of Patients With NV-HAP

Characteristics	NV-HAP, No. (%) (n = 32 792)
**Baseline oxygen device (before NV-HAP)**	
None	23 975 (73.1)
Nasal cannula	6766 (20.6)
Simple mask	604 (1.8)
Oxygen conserving device	33 (0.1)
Nonrebreather mask	243 (0.7)
High flow oxygen nasal cannula	559 (1.7)
BIPAP	617 (1.9)
**Highest oxygen device on day of NV-HAP**	
None	2475 (7.5)
Nasal cannula	12 803 (39.0)
Simple mask	1335 (4.1)
Oxygen conserving device	147 (0.4)
Nonrebreather mask	580 (1.8)
High flow oxygen nasal cannula	3898 (11.9)
BIPAP	3002 (9.2)
Ventilator	8557 (26.1)
Laboratory values on first day of NV-HAP	
White blood cell count (K/uL)	14 (10-18.6)
Hematocrit, %	29 (25-35)
Platelet count, K/uL	196 (130-179)
Creatinine, mg/dL	1.1 (0.8-1.9)
Glucose, mg/dL	161 (122-217)
Sodium, mmol/L	137 (133-140)
Albumin, g/dL	2.5 (2.0-3.0)
Alanine aminotransferase, IU/L	30 (18-61)
Total bilirubin, mg/dL	0.8 (0.5-1.4)
ICU 2 days before NV-HAP	8229 (25.1)
ICU on day of NV-HAP	20 123 (61.4)
Median days from admission to NV-HAP (IQR)	4 (2-7)
Median hospital length of stay, (IQR), d	17 (11-26)
Discharge disposition	
Home	12 449 (38.0)
Rehabilitation facility	1565 (4.8)
Skilled nursing facility	5783 (17.6)
Hospice	2629 (8.0)
Death	7361 (22.4)

Abbreviations: BIPAP, bilevel positive airway pressure; ICU, intensive care unit; NV-HAP, non–ventilator-associated hospital-acquired pneumonia.

To convert white blood cell count to ×10^9^/L, multiply by 0.001; hematocrit to proportion of 1.0, multiply by 0.01; platelet count to ×10^9^/L, multiply by 1.0; creatinine to μmol/L, 76.25; glucose to mmol/L, 0.0555; sodium to mmol/L, 1.0; albumin to g/L, multiply by 10; alanine aminotransferase to μkat/L, multiply by 0.0167; bilirubin to μmol/L, multiply by 17.104.

### Medical Record Review

On medical record review of 250 VA patient hospitalizations meeting electronic surveillance criteria, at least 1 reviewer, bedside clinician, or discharging clinician deemed NV-HAP to be present in 202 of 250 patients (81%); 168 patients (67%) met NHSN criteria. Both reviewers deemed 31 patients (12.4%) to lack an infiltrate, and 16 (6.4%) had an infiltrate that reviewers deemed either old or resolved. Interrater reliability between reviewers manually assessing for NV-HAP was low to moderate (simple agreement, 78%; Cohen κ, 0.55) and for applying CDC and NHSN criteria (simple agreement 75%; Cohen κ, 0.50). Among 62 cases where reviewers disagreed, the most common source of disagreement between reviewers was interpretation of chest imaging reports (37 [60%]). Complete medical record review results are available in eAppendix 4 of Supplement 1 and separate work.^13^

### Estimation of Attributable Mortality and Exploratory Analyses

**Figure 3.**
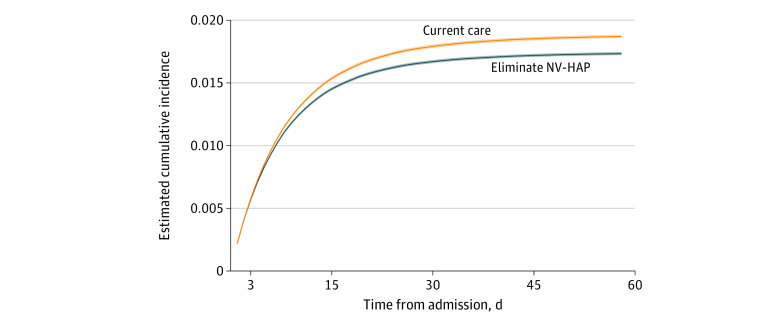
Incidence of Inpatient Death Up to 60 Days Follow-Up Under Hypothetical Elimination of NV-HAP vs Current Care Estimated cumulative incidence of inpatient death up to 60 days follow-up under hypothetical elimination of NV-HAP (blue) vs current care (orange). 95% CIs are shaded areas. NV-HAP indicates non–ventilator-associated hospital-acquired pneumonia.

Among the 4 038 974 hospitalizations included in the weighted analysis, the estimated cumulative risk of inpatient death for all patients by 60 days of follow-up was 1.87% under current care. Under the analysis corresponding to hypothetical elimination of NV-HAP, the estimated risk was 1.73% (absolute risk difference 0.14% [95% CI, 0.13%-0.14%]; relative risk ratio, 0.927 [95% CI, 0.92.5-0.928]) (Figure 3 and eAppendix 4 in Supplement 1). Differences in the cumulative risk of inpatient death vs alive discharge within 60 days were similar across stratifications by age, comorbidity burden, ICU status, service group, hospital size, teaching affiliation, and region, and when follow-up was limited to 30 days (eAppendix 4 in Supplement 1).

## Discussion

Among 284 diverse US hospitals from 2 large health care networks, approximately 1 in 200 admissions met electronic surveillance criteria for a possible NV-HAP event, of whom 22% died in-hospital, another 8% were discharged to hospice, and only 38% were discharged directly home. Incidence rates varied by the hospital from 1 per 300 admissions in the lowest quartile to 1 per 150 admissions in the highest quartile. We estimated that NV-HAP could be associated with up to 7.3% of hospital deaths. The incidence and crude individual mortality of NV-HAP was within the range reported in previous studies using point prevalence,^1^ manual,^30^ or semi-automated approaches.^31^ However, this study is the first to generate population-level estimates of the incidence, variation, outcomes, and attributable mortality of NV-HAP using an electronic clinical surveillance definition.

Measuring the true incidence of NV-HAP is inherently challenging. Pneumonia is a syndrome that lacks a reference standard for diagnosis. The signs of NV-HAP are neither sensitive nor specific.^32,33,34^ Respiratory cultures are obtained in less than a third of patients treated for NV-HAP despite guideline recommendations.^35,36^ Microbiologic confirmation of infection occurs in fewer than half of patients who undergo cultures.^37,38,39^ Clinical and radiographic interpretations vary between reviewers, correspond variably with histology, and clinical audits suggest that a third or more of patients treated for NV-HAP do not have pneumonia.^40,41,42,43,44^

Discharge diagnosis codes also do not provide reliable estimates of NV-HAP incidence and outcomes because they too are neither sensitive nor specific.^10,11^ Even cases identified using formal CDC criteria are inconsistently confirmed on external review.^7^ The electronic clinical criteria we applied in this study had a reasonable positive predictive value for clinically diagnosed pneumonia (81%) and moderate positive predictive value for CDC-NHSN criteria (67%). There is no reason to presume that the electronic surveillance definition is any more accurate than existing definitions since it relies on the same imperfect signs that clinicians and surveyors use to diagnose or track pneumonia. The advantage of electronic clinical criteria for NV-HAP instead is that they enable efficient surveillance in large numbers of diverse hospitals using consistent and reproducible criteria.

Most instances of NV-HAP affected clinically vulnerable patients. The median age of patients with NV-HAP was 69, most patients had multiple serious comorbidities, and one-fourth of NV-HAP events involved patients in intensive care units. The high-risk profile of the population who developed NV-HAP begs the question of the extent to which NV-HAP influenced these patients’ clinical trajectories: were these patients already at high risk of death during hospitalization even without NV-HAP, or did NV-HAP lead to otherwise avoidable adverse outcomes? We incorporated a rich array of clinical parameters into our analysis to account for confounding by patients’ baseline status and severity of illness using both fixed and time-varying parameters, including daily vital signs and laboratory measures. However, even after accounting for these factors, outcomes experienced by patients with NV-HAP remained worse than those without NV-HAP.

The high incidence and mortality rate associated with NV-HAP suggests it is an important hospital complication that warrants the development and testing of prevention programs. While there has been substantial work to date on defining best practices to prevent ventilator-associated pneumonia, there is very little consensus on how best to prevent NV-HAP.^45^ Potential strategies include enhanced oral care, mobilizing patients, minimizing the use of acid suppressants, and applying dysphagia precautions.^46,47,48,49^ Nurse-initiated oral care programs have suggested promising impacts on NV-HAP.^50,51^ However, these initiatives used manual medical record reviews or diagnosis codes to identify NV-HAP, a potential source of bias because clinical criteria are subjective and diagnosis codes are used variably by different clinicians and hospitals.^10,11^ Automated analyses of electronic clinical data may provide more consistent and efficient means to measure NV-HAP incidence and to track the impact of prevention programs. More broadly, our work provides a glimpse of how EHR-based surveillance has the potential to increase the number, breadth, granularity, consistency, and efficiency of quality and safety surveillance both within and between hospital systems.^52,53^

### Limitations

This study had limitations. There was imperfect concordance between NV-HAP surveillance criteria and confirmed pneumonia (a problem common to all clinical diagnostic and surveillance strategies due to the lack of a reference standard for pneumonia). Measurement of NV-HAP using the surveillance definition, although less subjective than diagnostic codes, could be influenced by the frequency with which vital signs and laboratory assays were measured, oxygen supplementation devices documented, variation in antibiotic prescribing and chest imaging thresholds between different clinicians and hospitals. We did not incorporate radiographic results into our surveillance definition, which may limit the definition’s specificity. Developments in natural language processing and image processing may make this more feasible and could improve the measure’s accuracy in the future.^54,55^ While our analysis of attributable mortality applied state-of-the-art analysis to detailed clinical data from 2 large national hospital networks, as in any observational analysis, there may remain unmeasured confounding, including changes in patient populations or care processes over time, particularly for the 7% of the population that coincided with the SARS-CoV-2 pandemic. We currently do not know what proportion of NV-HAP is truly preventable and hence the potential benefit of NV-HAP prevention programs. It will require prospective, real-world intervention studies, preferably randomized and multi-center, to determine the true extent to which NV-HAP can be prevented, the impact of doing so on mortality for both susceptible patients and hospital populations, and whether NV-HAP should be viewed as a measure of safety or quality. However, our study represents an early step forward by offering an estimate of incidence across diverse hospitals, associated mortality, and a generalizable surveillance method for NV-HAP.

## Conclusions

In a national study involving 2 large hospital networks, we report a robust estimate of the incidence of NV-HAP in US hospitals and its possible contribution to hospital-wide mortality. Our findings underscore the importance of developing and validating robust measurement tools to monitor NV-HAP incidence and the need to identify effective prevention strategies and assess the impact of prevention initiatives on patient and hospital-level outcomes.

## Notes

Supplement 1. **eAppendix 1.** Data Definitions Statistical Code **eAppendix 2. **Medical Record Review Guide **eAppendix 3.** Detailed Description of Weighted Analysis **eAppendix 4.** Results of Medical record Review and Secondary Analyses

Supplement 2.
Data Sharing Statement
