# Supplementary material for: Incidence and Outcomes of Non–Ventilator-Associated Hospital-Acquired Pneumonia in 284 US Hospitals Using Electronic Surveillance Criteria
Source: JAMA Netw Open. 2023 May 18;6(5):e2314185. doi: 10.1001/jamanetworkopen.2023.14185 (PMC10196873; doi:10.1001/jamanetworkopen.2023.14185)
Supplement: Supplement 1. — eAppendix 1. Data Definitions Statistical Code eAppendix 2. Medical Record Review Guide eAppendix 3. Detailed Description of Weighted Analysis eAppendix 4. Results of Medical record Review and Secondary Analyses [file jamanetwopen-e2314185-s001.pdf]

Supplemental Online Content

Jones BE, Sarvet AL, Ying J, et al. Incidence and outcomes of non–ventilator-associated hospital-acquired pneumonia in 284 US hospitals using electronic surveillance criteria. *JAMA Netw Open*. 2023;6(5):e2314185. doi:10.1001/jamanetworkopen.2023.14185

eAppendix 1. Data Definitions Statistical Code
eAppendix 2. Medical Record Review Guide
eAppendix 3. Detailed Description of Weighted Analysis
eAppendix 4. Results of Medical Record Review and Secondary Analyses

This supplemental material has been provided by the authors to give readers additional information about their work.

© 2023 Jones BE et al. *JAMA Network Open.*

eAppendix 1. Data Definitions Statistical Code

1. Surveillance definition of NV-HAP.

eTable 1. NV-HAP Surveillance Definition

Clinical features of NV-HAP	
Inclusion criteria	≥ 3 days hospitalization<br>Not receiving mechanical ventilation
Worsening oxygenation sustained for ≥2 calendar days	Drop in pulse oximetry from ≥95% on ambient air to <95% on ambient air, or<br>New initiation of supplemental oxygen, or<br>Escalation of supplemental oxygen:<br>o increase in flow rate of ≥3L/min for nasal cannula, ≥4L/min for face mask, or<br>o Escalation of oxgyen delivery device.<br>Escalation of devices was in accordance with the following hierarchy: mechanical ventilation > BIPAP > non-rebreather > high flow nasal cannula > oxygen conserving device > simple face mask > nasal cannula > none.
Fever, OR abnormal white blood cell count (WBC)	Temperature ≤36 or ≥38 °C, or<br>WBC <4,000 or ≥12,0000 cells/mm^3
Recognition/response by clinical team	
Performance of chest imaging	Evidence of order or procedure code for chest X-ray or computerized tomography of the chest
Initiation of new antibiotics	Administration of selected antimicrobials (e-Table 3) not previously administered in past 2 days and continued for ≥3 days (changes in antibiotics permitted during the 3 day period so long as each new agent was not used in the preceding 2 days).

© 2023 Jones BE et al. *JAMA Network Open.*

<u>Guidance on implementation:</u>
Among patients hospitalized for ≥ 3 days:

1. Oxygen saturation (SaO2), a measurement of the percentage of how much hemoglobin is saturated with oxygen. The normal range for SaO2 is 95-100%. Criteria to define respiratory deterioration are set out on the following Table 1 but they all follow a framework of looking for two days of stable oxygenation followed by 2 days of impaired oxygenation relative to the 2 baseline days. **Impaired oxygenation can be marked by a decrease in daily maximum SaO2 in a patient on room air alone, escalation from room air to the use of an oxygen delivery device, an increase in flow rate for a given oxygen delivery device, or escalation from a less efficient to a more efficient oxygen delivery device.** Details are specified in the eTable 2.

eTable 2. Criteria for increase in supplemental oxygen requirement

If patient has the following first device for ≥2 calendar days (including hospital days 1 and 2):	Respiratory deterioration is then deemed present **if last device on both the next two days** is greater than the first device for the two baseline days and the first + last device on the first baseline day:
Room air and SpO2 ≥95%	Room air with SpO2 < 95% or escalation to any oxygen delivery device, **sustained for≥2 calendar days**. The second calendar day of respiratory deterioration can either be the same **last device** as the first day of respiratory deterioration or a higher-level device (column 1 of this table arrays devices in order of escalation)
Room air and SpO2 ≥90%	Escalation to any new oxygen delivery device, sustained for ≥2 calendar days
Nasal cannula	Nasal cannula with increase in **median flow rate** by ≥3 liters per minute sustained for ≥2 calendar days, or escalation to any more intense device (face mask, non-rebreather, oxymizer, high-flow, BIPAP, ventilator) sustained for ≥2 calendar days
Simple mask	Increase in **median flow rate** by ≥4 liters per minute sustained for ≥2 calendar days, or escalation to any more intense device (non-rebreather, oxymizer, high-flow, BIPAP, ventilator) sustained for ≥2 calendar days

© 2023 Jones BE et al. *JAMA Network Open.*

Oxygen conserving device	Escalation to any more intense device (non-rebreather, high-flow, BIPAP, ventilator) sustained for ≥2 calendar days
Non-rebreather	Escalation to any more intense device (high-flow, BIPAP, ventilator) sustained for ≥2 calendar days
High-flow	Escalation to any more intense device (BIPAP, ventilator) sustained for ≥2 calendar days
BIPAP	Escalation to ventilator sustained for ≥2 calendar days
Ventilator	Exclude from analysis (study is focused on non-ventilated patients)

2. New antibiotics. We have two scenarios:
    1) We require new antibiotics started on the first day of respiratory deterioration or the day following respiratory deterioration and continued for at least 3 calendar days. First day of new antibiotics must be on hospital day 3 or greater.
    2) New antibiotics started on the first day of respiratory deterioration or the day following respiratory deterioration and continued for at least 3 calendar days. First day of new antibiotics must be on hospital day 3 or greater. We require new antibiotics started and continued for 1 or 2 days is also acceptable for definitions #2 and up if the patient dies on the day after the second day of respiratory deterioration. Different scenarios for timing of antibiotic start and date of death are:
        a) If day of death is on second day of impaired oxygenation then 2 days of antibiotics acceptable if antibiotics were stared on the first day of impaired oxygenation; 1 day of antibiotics acceptable if antibiotics were started on the second day of impaired oxygenation.
        b) If day of death is on the third day after the first day of impaired oxygenation then 2 days of antibiotics acceptable if antibiotics were stared on the first day of impaired oxygenation; 1 day of antibiotics acceptable if antibiotics were started on the second day of impaired oxygenation.
        c) If day of death is on the fourth day after the first day of impaired oxygenation then at least 3 days of antibiotics required if antibiotics were stared on the first day of impaired oxygenation; 2 days of antibiotics acceptable if antibiotics were started on the second day of impaired oxygenation.
3. Abnormal temperature. Abnormal temperature is defined as **max temperature ≥38°C or min temperature ≤36°C.** Fever had to be met on the first day of respiratory deterioration or the day after.
4. Abnormal WBC count. Abnormal WBC count is defined as **max WBC > 12,000 cells/mm^3 OR min WBC < 4,000 cells/mm^3**. Abnormal WBC had to be met on the first day of respiratory deterioration or the day after.
5. Chest imaging ordered on the first day of respiratory deterioration or the day after.

© 2023 Jones BE et al. *JAMA Network Open.*

e-Table 3. Select antimicrobials.	
Amikacin	Doripenem
Amoxicillin	Doxycycline
Amoxicilllin/clavulanate	Eravacycline
Ampicillin	Erithromycin
Ampicillin-sulbactam	Ertapenem
Azithromycin	Gatifloxacin
Aztreonam	Gentamicin
Carbenicillin	Imipenem
Cefaclor	Imipenem/Relebactam
Cefadroxil	Lefamulin
Cefazolin	Levofloxacin
Cefdinir	Linezolid
Cefepime	Meropenem
Cefiderocol	Meropenem/Vaborbactam
Cefixime	Metronidazole
Cefotaxime	Minocycline
Cefotetan	Moxifloxacin
Cefoxitin	Nafcillin
Cefpodoxime	Omadacycline
Cefproxil	Oritavancin
Ceftaroline	Oseltamivir
Ceftazidime	Oxacillin
Ceftazidime-avibactam	Penicillin
Ceftolozane-tazobactam	Peramivir
Ceftriaxone	Piperacillin
Cefuroxime	Piperacillin-tazobactam
Cephalexin	Plazomicin
Cilastin/Imipemen	Polymyxin B
Ciprofloxacin	Tedizolid
Clarithromycin	Telavancin
Clavulanate/ticarcillin	Telithromycin
Clindamycin	Tetracycline
Colistin	Ticarcillin
Dalbavancin	Tigecycline
Dalfopristin/quinopristin	Tobramycin
Delafloxacin	Trimethoprim-sulfamethoxazole
Dicloxacillin	Vancomycin - IV only

© 2023 Jones BE et al. *JAMA Network Open.*

**eTable 4. Guidance on how to handle patients who die before they can receive 3 days of antibiotics**				
**Hospital Day***	**Oxygenation**	**Antibiotics Scenario 1**	**Antibiotics Scenario 2**	
1	Baseline			
2	Baseline			
3	Impaired	Abx day 1		
4	Impaired	Abx day 2	Abx day 1	If day of death is on second day of impaired oxygenation then 2 days of antibiotics acceptable if antibiotics were stared on the first day of impaired oxygenation; 1 day of antibiotics acceptable if antibiotics were started on the second day of impaired oxygenation.
5		Abx day 3	Abx day 2	If day of death is on the third day after the first day of impaired oxygenation then 2 days of antibiotics acceptable if antibiotics were stared on the first day of impaired oxygenation; 1 day of antibiotics acceptable if antibiotics were started on the second day of impaired oxygenation.
6			Abx day 3	If day of death is on the fourth day after the first day of impaired oxygenation then at least 3 days of antibiotics required if antibiotics were stared on the first day of impaired oxygenation; 2 days of antibiotics acceptable if antibiotics were started on the second day of impaired oxygenation.

*Hospital day can be any hospitalization day t.

© 2023 Jones BE et al. *JAMA Network Open.*

eTable 5. Mapping of device type

**Device Name**	**Map to**
Aerosol mask	Simple mask
Bag-valve Mask	Ventilator
Bi-PAP	BIPAP
Blow-by	Simple mask
CPAP	Nasal cannula
Face tent	Simple mask
High flow face mask	High flow
High flow nasal cannula	High flow
Hood	Simple mask
Nasal cannula	Nasal cannula
Non-rebreather mask	Non-rebreather
None (Room air)	None
Oxygen conserving device	Oxygen conservation device
Partial rebreather mask	Simple mask
Simple mask	Simple mask
T-Piece	Ventilator
Trach mask	Simple mask
Ventilator	Ventilator
Venturi mask	Simple mask
Transtracheal catheter	Simple mask
Pulse dose device	Pulse device

Data definitions were programmed in SAS, available in "NV-HAP Surveillance Definitions and statistical code" (2022), GitHub repository, https://github.com/caramckenna/NVHAP

eAppendix 2. Medical Record Review Guide

The following information was abstracted by reviewers:

1. Presence of *unstructured* features included in the CDC-NHSN PNEU criteria (Box 1)

2. Presence of *unstructured features* included in the NVHAP event criteria (Box 2)

3. Presence of assertion of pneumonia per treating clinician documentation within 2 calendar days of index date.

4. The treating clinician's perceptions (Including: admission diagnosis, presence of a clinical deterioration, clinician recognition of a deterioration, perceived cause, and presence of pneumonia in discharge diagnoses)

5. Reviewers' assessment of the cause of clinical deterioration and whether or not NVHAP was present

© 2023 Jones BE et al. *JAMA Network Open.*

Box 1 – Adapted CDC-NHSN PNU1 Criteria.

NHSN criteria were adapted slightly to provide a more specific definition of oxygen deterioration (Box 2) and infiltrate on chest imaging (a).

See NHSN Patient Safety Component Manual Ch. 6 Table 1 (pdf page 94) summarized below. Items 1-3 must be present and occur during the specified timeframes.

1. Chest imaging (≥ 2 images within 7 days*).
    a. Initial qualifying image must occur during the infection window period (IWP)**
    b. Imaging study must demonstrate infiltrate, consolidation, or cavitation that is new and persistent, or progressive and persistent.*** [a]

2. At least *one* of the following (must occur during IWP**)
    a. Fever (>38.0°C or >100.4°F)
    b. Leukopenia (≤4000 WBC/mm3) **OR** Leukocytosis (≥12,000 WBC/mm3)
    c. For adults ≥ 70 years old, altered mental status with no other recognized cause

3. At least *two* of the following (Must occur during NVHAP days 1-2)
    a. New onset of purulent sputum, change in sputum character, increased respiratory secretions, or increased suctioning requirements
    b. New or worsening cough, dyspnea, or tachypnea (RR>25)
    c. Rales ("crackles") or bronchial breath sounds (including "rhonchi")
    d. Worsening gas exchange (see Box 2, item 1)

* If a patient does **not** have underlying cardiopulmonary disease, then only 1 chest image is required. However, when multiple imaging test results are available during the IWP, persistence of imaging test evidence of pneumonia is a requirement for all patients (even those without underlying cardiopulmonary disease).

** Infection Window Period (IWP): Defined as 7-day window including Index Date and 3 days before and 3 days after Index Date (Table 2 below). CDC-PNEU criteria including the first positive imaging study must occur during this timeframe to meet criteria.

*** An infiltrate (see [a] for definition) that is new on earlier image and resolves on later image within 7 days does not qualify for pneumonia.

Index Date

Table 2: Infection Window Period

w Period		**3 days before**
	Date of first positive diagnostic test that is used as an element of the site-specific criterion	

Box 2 – NVHAP Surveillance Event Definition

See "Definition 7" from Ji et al JAMA Network Open, (2019), e1913674, 2(10). Item 1 (deoxygenation) is used to identify a possible candidate event and date (NVHAP Day 1). Items 2 – 4 must occur within 2 days (NVHAP Day 1 – 2). Items 1 – 4 required.

1. Deoxygenation sustained for ≥2 days. Defined as *any* of:
    a. Drop in saturations from ≥95% on ambient air to <95% on ambient air
    b. Initiation of supplemental oxygen
    c. Escalation of supplemental oxygen (increase in flow rate or device: RA < NC < facemask < HFNC/non-invasive mechanical ventilation [BiPAP] < mechanical ventilation)

2. Fever (>38.0°C or >100.4°F) **OR** Leukopenia (≤4000 WBC/mm3) **OR** Leukocytosis (≥12,000 WBC/mm3)

3. Initiation of new antibiotics that were not given in the previous 2 calendar days

4. Chest imaging obtained (CXR or CT scan)

Instructions for Chart Review:

Information Field	Instructions for Field Collection
**Step 1:** Open the chart in Capri using site, last name, and last 4 of SSN	
Site<br>Last Name, First Name<br>Last4ssn<br>Age<br>Index Date<br>Admit Date, Discharge Date	This information is prepopulated for each case
Reviewer	Confirm that the reviewer selected matches your initials
Date Reviewed	Enter the date that you (the reviewer) did the review for this particular case
**Step 2:** Review notes (filter by date from Admit to Discharge) and fill fields below as you go<br>Open the clinical documents tab and filter to view notes from Admit date to discharge date<br>- review H&P<br>- review notes from date of event and up to 2 days after (MD > consults > RN > RT > other)<br>- review discharge summary	
Admit Dx	Working admit Dx asserted by primary team during 2 day window (hospital day 1 or 2)
Pneumonia diagnosed on Admit?	Check box if there is clinical assertion of pneumonia by treating team within 2 calendar days of hospitalization (hospital day 1 or 2)
Underlying cardiopulmonary disease	Check box if there is underlying chronic cardiopulmonary disease (e.g. COPD, CHF, ARDS, ILD, lung malignancy, etc.). Do <u>not</u> include comorbidities such as HTN, CAD, asthma that would not produce imaging findings that could mimic pneumonia.
Clinical deterioration/ setback present?	Check box if there is evidence of any incident that negatively influences patient's clinical trajectory associated with index date
Symptom onset >2 days from admit	This pertains to symptoms included in CDC/NHSN list of symptoms or that could be clinically related to a diagnosis of pneumonia (ie **not** diarrhea or abdominal pain)
Change in sats or device/flow?	Review notes on NVHAP days 1-2 for evidence of <u>any</u> of the following:<br>a. Drop in saturations from ≥95% on ambient air to <95% on ambient air

© 2023 Jones BE et al. *JAMA Network Open.*

	b. Initiation of supplemental oxygen<br>c. Escalation of supplemental oxygen (increase in flow rate or device: Room air < nasal cannula < facemask < high-flow nasal cannula ~ non-invasive mechanical ventilation [BiPAP] < mechanical ventilation)
Deterioration perceived by clinician?	Did the clinicians assert there was a clinical change (of any kind)
Clinician leading dx	What does the clinician attribute the declining oxygenation event to (ex: "pulmonary edema")
Clinician diagnosis of pneumonia	Check box if treating clinician asserts diagnosis of pneumonia within 2 days following index date (NVHAP day 1-2). Do they SAY it, not "were they THINKING it?"
Discharge dx pneumonia	check box if pneumonia is listed anywhere in discharge summary regardless of type
Clinician perception Summary	Briefly summarize clinician's thought process in formulating treatment plan. ex: "thought to be pulmonary edema vs HAP"
For the following clinical criteria, presence is "yes" if there was an occurrence any time **during NVHAP days 1-2** (event date is NVHAP day 1)	
Fever	Prepopulated if present during IWP. "yes" if temperature >38.0°C (100.4°F)
WBC	Prepopulated if present during IWP. "yes" if WBC ≤4000 or ≥12,000
AMS	check box if there is evidence of altered mental status (regardless of perceived contributing factors)
Change in sputum?	Includes new onset or change in character, increased respiratory secretions, or increased suctioning requirements
Cough/dyspnea	New onset or worsening cough, dyspnea, or shortness of breath
Abnormal breath sounds	Abnormal lung sounds including Rales, crackles, bronchial breath sounds, rhonchi. Example words that DO NOT count include "diminished" or "coarse".
Tachypnea (RR>25)	Prepopulated if present during IWP. "yes" if respiratory rate was >25 in vitals

© 2023 Jones BE et al. *JAMA Network Open.*

New/change in antibiotics	Prepopulated. “yes” if there was initiation of or change in antibiotics during NVHAP days 1-2 that was not given during the previous 2 calendar days<br><br>* If unclear: go to the Meds tab, click Med Admin History (BCMA), right click to search by date range, search 2 days prior to index date and 3 days including/after index date (NVHAP days -2, -1, 1, 2, and 3) for evidence of new antibiotics or a change in antibiotics on NVHAP days 1-2.
**Step 3:** Review imaging reports<br>- review chest imaging on NVHAP day 1 and 3 days before and after (NVHAP days -3, -2,-1, **1**, 2, 3, 4). The “infection window period”.<br>- if an infiltrate is identified, review chest imaging up to 7 days after the first positive image for evidence of resolution	
Chest Imaging Obtained?	Click box if there was *any* type of chest imaging (Chest X-Ray or CT Chest) on NVHAP days -3 to +4
2+ images obtained?	Click box if there were Two or more POSITIVE serial chest imaging results Within 7 days, starting at the first day of chest imaging within the infection window period. Note that if the initial image was POSITIVE and subsequent images note that the finding was improved but not resolved, that this still counts as a POSITIVE follow-up imaging study.
Chest imaging result	Select the appropriate image interpretation by the clinical team (either in clinical notes OR in radiology report.<br>• If clinical team asserts infiltrate present but radiology report does not, this counts as an infiltrate).<br><br>• Options: “no infiltrate";"new & persistent/progressive infiltrate";"new infiltrate no comparison";"old infiltrate"; “infiltrate that resolved”<br><br>**[a] Guidelines for reports to qualify for “infiltrate”:**<br>1. First review the findings [NOT the indication] and look for any note of the following (Must have at least 1)<br>o Airspace disease<br>o Bronchogram, Bronchopneumonia<br>o Consolidation, Consolidative process<br>o Density

© 2023 Jones BE et al. *JAMA Network Open.*

- Increased interstitial markings, Increased lung markings
- Infiltrate, Infiltration, Infiltrative process, Positive infiltrate
- Inflammation, Inflammatory process
- Interstitial pneumonia, Interstitial process
- Haziness
- Opacity, Opacification
- Patchiness
- Pneumonitis
- Reticulonodular pattern

2. Review the impression for any mention of pneumonia OR infection

Interpretation: “Positive” for infiltrate IF

- There is ANY mention of pneumonia or infection
- OR There is at least 1 finding in (1) AND no alternative explanation

Positive examples:

- Findings list “consolidation” and Impression includes “possible pneumonia”
- Findings list “interstitial markings” but impression not given
- Findings list “haziness” and impression lists “atelectasis, cannot rule out infectious process”

Negative examples:

- Findings list “interstitial markings” and impression lists “consistent with pulmonary edema”

Step 4: Determine whether criteria was met

© 2023 Jones BE et al. *JAMA Network Open.*

CDC PNEU?	Use CDC PNEU criteria met (Box 1 adaptation). Must meet all of items 1 - 4<br><br>1. Chest imaging Obtained AND New & progressive/persistent infiltrate<br><br>2. 2+ images IF Underlying Cardiopulmonary dx<br><br>3. At least *one* of the following<br>a. Fever<br>b. WBC<br>c. AMS AND Age > 70<br><br>4. At least *two* of the following (a – d)<br>a. Change in Sputum<br>b. Cough/dyspnea OR tachypnea (RR>25)<br>c. Abnormal breath sounds<br>d. Change in sats/device/flow
NVHAP candidate definition (def7) met?	Use NVHAP Surveillance Event Definition (Box 1). Must meet all 4 criteria during NVHAP days 1-2 (named fields must be ticked):<br><br>1. Change in sats/device/flow<br>2. Fever OR WBC<br>3. New/change in Antibiotics<br>4. Chest Imaging Obtained
**Step 5:** Reviewers assessment	
NVHAP per Reviewer?	Select from "No";"Possible";"Probable"
Reviewer leading diagnosis	Diagnosis that reviewer thinks is responsible for change in patient's clinical status
Reviewer narrative	Briefly summarize pertinent events of hospitalization, can include details of pertinent studies and treatments and clinical course
Bonus: assess whether the patient was intubated or not in relation to the event	

© 2023 Jones BE et al. *JAMA Network Open.*

Intubated <= 2 days pre/post index date?	Check box if patient was intubated during 2 day window including day prior to index date, index date, and day following index date (NVHAP days -1, 1, 2)
Vent Start Date	Enter ventilator start date
Vent Stop Date	Enter date of first extubation after initiation of this intubation
Vent Narrative	Free text any additional pertinent ventilator narrative

© 2023 Jones BE et al. *JAMA Network Open.*

eAppendix 3. Detailed Description of Weighted Analysis

To estimate the risk of inpatient death and alive discharge under hypothetical elimination of NV-HAP, we applied an inverse probability weighted Aalen-Johansen estimator of the cause-specific cumulative incidence for competing events; discharge is a competing event for hospital mortality (and vice versa) in the sense that once one event occurs, the other subsequently cannot for that particular hospitalization (Aalen ref, Young et al., 2020). The cause-specific cumulative incidence for hospital mortality by a particular follow-up day of that hospitalization is simply the proportion of the original baseline population who died by that day of their hospitalization. Individuals who are discharged prior to that follow-up time, by definition, did not die by that day of hospitalization and therefore contribute to the denominator but not the numerator of that proportion. The Aalen-Johansen estimator was originally developed to estimate a cause-specific cumulative incidence for censored data. Here we leveraged an extension of this method for "artificially censored" data developed for estimating counterfactual outcome distributions under hypothetical interventions in observational studies.[22]

We artificially censored an individual's follow-up information as soon as their data became inconsistent with an intervention that would theoretically eliminate NV-HAP: more specifically, we censored individuals meeting criteria for NV-HAP on the first day they met those criteria. The Aalen-Johansen estimator of the cause-specific cumulative incidence for an event of interest (e.g. hospital death) by a given time t is a function of the estimated time-varying cause-specific hazards of that event of interest and any competing events (e.g. when the event of interest is hospital death, discharge alive is a competing event) up to that time. When there is no censoring in the data, then this estimator will be equivalent to just calculating a proportion with the denominator taken to be the number of individuals in the original study population and numerator taken to be the number experiencing the event of interest by t. In censored data, this estimator, which does not account for covariates, will rely on assumptions on the censoring process that will be violated if there are common causes of the event of interest and censoring events. [22]

Because the censoring event occurs whenever an individual meets criteria for NV-HAP, such an assumption is unreasonable. We therefore applied a weighted version of the traditional Aalen-Johanssen estimator that can adjust for possible common causes or measured proxies of common causes of censoring and the event of interest, where person-time weights are applied to the time-varying cause-specific hazards which are, in turn, used to calculated an adjusted estimate of the cause-specific cumulative incidence or "risk" of the event of interest by a given time. These inverse probability weights (IPW) depend on an estimate of the probability of remaining free of NV-HAP (i.e. remaining uncensored) by each day t conditional on the assumed baseline and time-varying "confounders". We refer to these as "confounders" because in our case, censoring status is entirely a function of exposure (i.e. NV-HAP) status. In turn, the probability of remaining uncensored by t is entirely a function of a special case of a time-varying propensity score in this case.

On all follow-up days t in which a person was still uncensored and still hospitalized alive ("at risk"), they received a nonzero inverse probability weight that depends on estimates of their propensity scores up to t; that is the chance of NV-HAP on each day conditional on that

© 2023 Jones BE et al. *JAMA Network Open.*

individual's confounder history. For a censored individual, their person-time is weighted as zero from that point forward. However, this individual will contribute person-time information to the overall risk estimate prior to that time (during their "uncensored" time).

- We estimated propensity scores using a pooled over time logistic regression model with dependent variable NV-HAP status on a particular day and independent variables follow-up day and past values of time-fixed and time-varying confounders. Continuous variables for age and routine laboratory were included in the propensity models as restricted cubic splines; variables for non-routine laboratory results were categorized as following:

Lab	Result Range	Code as
ALT	Missing/unmeasured	0
	0-50	1
	51-99	2
	100-199	3
	200-499	4
	≥500	5
Tbili	Missing/unmeasured	0
	0-1.0	1
	1.1-1.9	2
	2.0-2.9	3
	3.0-4.9	4
	5.0-9.9	5
	≥=10	6
Albumin	Missing/unmeasured	0
	0-0.9	1
	1.0-1.9	2
	2.-2.9	3
	3.0-3.5	4
	≥3.6	5

The same approach was used to estimate the risk of either outcome under current care at a given time but with weights set to one for all hospitalizations and follow-up times in the original uncensored data (again, equivalent, to just nonparametrically estimating the cumulative proportion of events by a given time). Risk ratios and risk differences for both outcomes were then calculated by taking the ratio and difference, respectively, of the hypothetical intervention risk estimate versus that under current care. We generated 95% confidence intervals using a nonparametric bootstrap to resample hospitalizations with replacement 500 times. Secondary analyses stratified these analyses by age (≤65 or >65 years), service group (medical, surgical, cardiology, neuroscience, oncology, and other), ICU status on hospital day 3, Elixhauser comorbidity index quartile, hospital number of beds, region, and hospital teaching status. Hospital-level point estimates and bootstraps were computed separately for VA and HCA in order to maintain data security but then combined using weighted averages, with site-specific weights proportional to sample size (i.e., the number of unique hospitalizations associated with each data source). This procedure ensured data security protocols were followed but also that point estimates and 95% confidence intervals for the overall combined population were identical

© 2023 Jones BE et al. *JAMA Network Open.*

to those we would have obtained had we been able to analyze the VA and HCA data from a single pooled data set.
All statistical code and information on data handling is available in GitHub ()

© 2023 Jones BE et al. *JAMA Network Open.*

eAppendix 4. Results of Medical Record Review and Secondary Analyses

e-Table 6. Chart review validation among 250 cases meeting surveillance criteria for NV-HAP.				
Definition	Charts Positive per Any Reviewer n (% total)	Charts Positive per Both Reviewers n (% total)	Inter-Rater Agreement	Cohen's Kappa (CI)
NV-HAP per clinician reviewer	178 (71%)	124 (50%)	78%	0.55 (0.45 – 0.66)
CDC-NHSN criteria for nosocomial pneumonia met	168 (67%)	106 (42%)	75%	0.50 (0.39 – 0.61)
NV-HAP per treating clinician	151 (60%)	106 (42%)	82%	0.64 (0.55 – 0.74)
Pneumonia in discharge summary	122 (49%)	87 (35%)	86%	0.71 (0.63 – 0.80)

© 2023 Jones BE et al. *JAMA Network Open.*

e-Table 7. Risk of inpatient mortality and discharge at 60 days with elimination of NV-HAP versus existing care. Results are reported for overall population and stratified by age, service group, ICU, Elixhauser quartile, bed size, region, and teaching status.									
		Death at 60 days				Discharge at 60 days			
	Total n	Eliminate NV-HAP	Existing care	Risk ratio	Risk difference	Eliminate NV-HAP	Existing care	Risk ratio	Risk difference
Primary analysis	4038974	0.0173 (0.0172-0.0174)	0.0187 (0.0186-0.0188)	0.9268 (0.9245-0.9284)	-0.0014 (-0.0014 – -0.0013)	0.9804 (0.9803 – 0.9805)	0.9789 (0.9788 – 0.9791)	1.0015 (1.0015 – 1.0016)	0.0015 (0.0014 - 0.0015)
Age									
<65	1612976	0.0099 (0.0098-0.0101)	0.0109 (0.0108-0.0111)	0.912 (0.9074-0.9162)	-0.001 (-0.001 – -9e-04)	0.9877 (0.9876 – 0.9879)	0.9867 (0.9865 – 0.9868)	1.0011 (1.001 – 1.0012)	0.0011 (0.001 - 0.0011)
>=65	2425998	0.0223 (0.0221-0.0224)	0.0239 (0.0237-0.0241)	0.9313 (0.9288-0.933)	-0.0016 (-0.0017 – -0.0016)	0.9755 (0.9753 – 0.9757)	0.9737 (0.9736 – 0.974)	1.0018 (1.0017 – 1.0019)	0.0017 (0.0017 - 0.0018)
Service group									
Cardiology	131805	0.0255 (0.0247-0.0264)	0.0278 (0.027-0.0288)	0.9153 (0.9059-0.924)	-0.0024 (-0.0026 – -0.0021)	0.9729 (0.9721 – 0.9738)	0.9705 (0.9695 – 0.9714)	1.0026 (1.0023 – 1.0028)	0.0025 (0.0022 - 0.0028)
Medicine	2616022	0.0203 (0.0202-0.0205)	0.0218 (0.0217-0.0221)	0.9284 (0.9259-0.9303)	-0.0016 (-0.0016 – -0.0015)	0.9773 (0.9771 – 0.9774)	0.9756 (0.9754 – 0.9758)	1.0017 (1.0016 - 1.0018)	0.0017 (0.0016 - 0.0017)
Surgery	1045813	0.0099 (0.0097-0.01)	0.0108 (0.0106-0.011)	0.9146 (0.9095-0.9206)	-9e-04 (-0.001 – -9e-04)	0.988 (0.9879 – 0.9883)	0.9869 (0.9868 - 0.9872)	1.0011 (1.001 - 1.0012)	0.0011 (0.001 - 0.0011)
Neurology	35808	0.0264 (0.0254-0.0288)	0.0288 (0.0278-0.0313)	0.9161 (0.8972-0.9346)	-0.0024 (-0.0031 - -0.0019)	0.9718 (0.9694 – 0.9728)	0.9693 (0.9667 - 0.9704)	1.0026 (1.0021 - 1.0033)	0.0025 (0.002 - 0.0032)
Oncology	29387	0.0224 (0.0205-0.0239)	0.0245 (0.0225-0.0259)	0.9174 (0.8956-0.938)	-0.002 (-0.0026 – -0.0015)	0.9735 (0.9721 – 0.9755)	0.9711 (0.9696 - 0.9733)	1.0024 (1.0018 - 1.003)	0.0024 (0.0018 - 0.003)
Other	180139	0.0095 (0.0097-0.0107)	0.0096 (0.0097-0.0107)	0.9967 (0.9938-0.9994)	0 (-1e-04 – 0)	0.9892 (0.988 – 0.989)	0.9891 (0.988 - 0.989)	1 (1 – 1.0001)	0 (0 – 1e-04)
ICU: No	3404201	0.0111	0.0122	0.916	-0.001	0.9871	0.986	1.0011	0.0011

© 2023 Jones BE et al. *JAMA Network Open.*

		(0.011-0.0112)	(0.012-0.0122)	(0.9129-0.9186)	(-0.0011 – -0.001)	(0.987 – 0.9872)	(0.9859 - 0.9861)	(1.0011 - 1.0011)	(0.001 - 0.0011)
ICU: Yes	634773	0.0507 (0.0502-0.0513)	0.0538 (0.0533-0.0544)	0.9426 (0.9397-0.945)	-0.0031 (-0.0033 – -0.003)	0.9444 (0.9439 – 0.945)	0.941 (0.9404 – 0.9416)	1.0036 (1.0035 - 1.0038)	0.0034 (0.0033 - 0.0036)
Elixhauser quartile									
Q1	1132108	0.0025 (0.0024-0.0026)	0.0026 (0.0025-0.0027)	0.9545 (0.9476-0.9624)	-1e-04 (-1e-04 – -1e-04)	0.9962 (0.9961 – 0.9964)	0.9961 (0.996 - 0.9962)	1.0001 (1.0001 - 1.0002)	1e-04 (1e-04 - 2e-04)
Q2	1113632	0.0078 (0.0076-0.0079)	0.0083 (0.0081-0.0085)	0.9358 (0.9307-0.9413)	-5e-04 (-6e-04 – -5e-04)	0.9908 (0.9906 – 0.9909)	0.9902 (0.99 - 0.9904)	1.0006 (1.0005 - 1.0006)	6e-04 (5e-04 - 6e-04)
Q3	845756	0.018 (0.0177-0.0183)	0.019 (0.019-0.0196)	0.9326 (0.9283-0.9366)	-0.0013 (-0.0014 – -0.0012)	0.9800 (0.9797 – 0.9803)	0.9786 (0.9783 - 0.9789)	1.0014 (1.0013 - 1.0015)	0.0014 (0.0013 - 0.0015)
Q4	947478	0.0459 (0.0456-0.0464)	0.0497 (0.0494-0.0502)	0.9231 (0.9202-0.9254)	-0.0038 (-0.004 – -0.0037)	0.9495 (0.949 – 0.9499)	0.9454 (0.9449 - 0.9457)	1.0044 (1.0042 - 1.0045)	0.0041 (0.004 - 0.0043)
Bed size									
1-99	738778	0.0167 (0.0164-0.0169)	0.0175 (0.0172-0.0178)	0.9533 (0.9498-0.957)	-8e-04 (-9e-04 – -7e-04)	0.9811 (0.9809 – 0.9815)	0.9803 (0.98 - 0.9806)	1.0009 (1.0008 - 1.0009)	9e-04 (8e-04 - 9e-04)
100-199	1478289	0.0175 (0.0172-0.0177)	0.0187 (0.0185-0.0189)	0.9329 (0.9299-0.9358)	-0.0013 (-0.0013 – -0.0012)	0.9798 (0.9796 – 0.9801)	0.9784 (0.9783 - 0.9787)	1.0014 (1.0013 - 1.0015)	0.0014 (0.0013 - 0.0014)
199-299	803522	0.0173 (0.017-0.0175)	0.0188 (0.0184-0.019)	0.9212 (0.9173 – 0.9258)	-0.0015 (-0.0016 – -0.0014)	0.9806 (0.9804 – 0.9809)	0.979 (0.9787 - 0.9794)	1.0016 (1.0015 - 1.0017)	0.0016 (0.0015 - 0.0017)
Region									
Midwest	630438	0.0163 (0.016 – 0.0166)	0.0173 (0.017-0.0177)	0.9401 (0.9354 – 0.9443)	-0.001 (-0.0011 – -0.001)	0.9823 (0.9819 – 0.9826)	0.9812 (0.9808 – 0.9815)	1.0011 (1.0011 - 1.0012)	0.0011 (0.001 - 0.0012)
Northeast	308521	0.0187 (0.0182–0.0192)	0.0198 (0.0193-0.0203)	0.9417 (0.9357 – 0.9475)	-0.0012 (-0.0013 – -0.001)	0.978 (0.9775 – 0.9785)	0.9767 (0.9762 – 0.9772)	1.0013 (1.0012 - 1.0015)	0.0013 (0.0012 - 0.0014)
South	2369521	0.0171 (0.0169–	0.0186 (0.0184-	0.9215 (0.9186 –	-0.0015 (-0.0015 –	0.9808 (0.9807 –	0.9793 (0.9791 –	1.0016	0.0016

© 2023 Jones BE et al. *JAMA Network Open.*

		0.0172)	0.0187)	0.9241)	-0.0014)	0.981)	0.9795)	(1.0015 - 1.0016)	(0.0015 - 0.0016)
West	689748	0.0168 (0.0166–0.0172)	0.0182 (0.018-0.0186)	0.9244 (0.9191 - 0.9297)	-0.0014 (-0.0015 – -0.0013)	0.9799 (0.9795 – 0.9802)	0.9784 (0.978 – 0.9787)	1.0015 (1.0014 - 1.0016)	0.0015 (0.0014 - 0.0016)
No	956499	0.0155 (0.0153–0.0158)	0.0169 (0.0167-0.0172)	0.9193 (0.9145 - 0.9235)	-0.0014 (-0.0014 – -0.0013)	0.983 (0.9827 – 0.9833)	0.9816 (0.9813 – 0.9818)	1.0015 (1.0014 - 1.0016)	0.0014 (0.0014 - 0.0015)
Yes	3082475	0.0179 (0.0177-0.018)	0.0193 (0.0191-0.0194)	0.9289 (0.9266 - 0.9308)	-0.0014 (-0.0014 – -0.0013)	0.9796 (0.9794 – 0.9798)	0.9781 (0.9779 – 0.9783)	1.0015 (1.0015 - 1.0016)	0.0015 (0.0014 - 0.0015)

© 2023 Jones BE et al. *JAMA Network Open.*

e-Table 8. Risk of inpatient mortality and discharge at 30 days with elimination of NV-HAP versus existing care. Results are reported for overall population and stratified by age, service group, ICU, Elixhauser quartile, bed size, region, and teaching status.									
		Death at 30 days				Discharge at 30 days			
	Total n	Eliminate NV-HAP	Existing care	Risk ratio	Risk difference	Eliminate NV-HAP	Existing care	Risk ratio	Risk difference
Primary analysis	4038974	0.0166 (0.0164 - 0.0167)	0.0178 (0.0176 - 0.0179)	0.9328 (0.9307 - 0.9344)	-0.0012 (-0.0012 - -0.0012)	0.9712 (0.9711 - 0.9715)	0.9694 (0.9693 - 0.9696)	1.0019 (1.0018 - 1.0019)	0.0018 (0.0018 - 0.0019)
Age: <65	1612976	0.0094 (0.0093 - 0.0096)	0.0102 (0.0101 - 0.0104)	0.9199 (0.9155 - 0.9242)	-8e-04 (-9e-04 - -8e-04)	0.9781 (0.9779 - 0.9783)	0.9767 (0.9764 - 0.9769)	1.0015 (1.0014 - 1.0015)	0.0014 (0.0014 - 0.0015)
Age: >=65	2425998	0.0213 (0.0212 - 0.0215)	0.0228 (0.0226 - 0.023)	0.9366 (0.9341 - 0.9384)	-0.0014 (-0.0015 - -0.0014)	0.9666 (0.9665 - 0.967)	0.9646 (0.9644 - 0.9649)	1.0022 (1.0021 - 1.0022)	0.0021 (0.002 - 0.0022)
Service group: Cardiology	131805	0.0247 (0.0239 - 0.0256)	0.0268 (0.026 - 0.0277)	0.9202 (0.9106 - 0.9293)	-0.0021 (-0.0024 - -0.0019)	0.9644 (0.9634 - 0.9654)	0.9615 (0.9604 - 0.9625)	1.003 (1.0028 - 1.0034)	0.0029 (0.0026 - 0.0032)
Service group: Medicine	2616022	0.0194 (0.0193 - 0.0196)	0.0208 (0.0206 - 0.021)	0.9339 (0.9314 - 0.936)	-0.0014 (-0.0014 - -0.0013)	0.9679 (0.9677 - 0.9681)	0.9659 (0.9657 - 0.9661)	1.0021 (1.002 - 1.0021)	0.002 (0.0019 - 0.0021)
Service group: Surgery	1045813	0.0093 (0.0091 - 0.0095)	0.0101 (0.0099 - 0.0103)	0.9242 (0.9191 - 0.9301)	-8e-04 (-8e-04 - -7e-04)	0.9798 (0.9798 - 0.9803)	0.9784 (0.9783 - 0.9788)	1.0015 (1.0014 - 1.0016)	0.0015 (0.0014 - 0.0016)
Service group: Neuroscience	35808	0.0259 (0.025 - 0.0284)	0.0283 (0.0274 - 0.0308)	0.9171 (0.8974 - 0.9351)	-0.0023 (-0.003 - -0.0019)	0.9606 (0.9578 - 0.9618)	0.9574 (0.9544 - 0.9588)	1.0033 (1.0027 - 1.004)	0.0031 (0.0026 - 0.0038)
Service group: Oncology	29387	0.0205 (0.0186 - 0.0219)	0.0222 (0.0203 - 0.0236)	0.9242 (0.9008 - 0.9437)	-0.0017 (-0.0022 - -0.0012)	0.9485 (0.9458 - 0.951)	0.9455 (0.9428 - 0.948)	1.0032 (1.0025 - 1.0039)	0.003 (0.0024 - 0.0037)
Service group: Other	180139	0.0094 (0.0095 - 0.0105)	0.0094 (0.0096 - 0.0105)	0.9967 (0.9938 - 0.9994)	0 (-1e-04 - 0)	0.9804 (0.9791 - 0.9804)	0.9803 (0.979 - 0.9803)	1.0001 (1 - 1.0001)	1e-04 (0 - 1e-04)
ICU: No	3404201	0.0106 (0.0105 - 0.0107)	0.0115 (0.0114 - 0.0116)	0.9217 (0.9186 - 0.9242)	-9e-04 (-9e-04 - -9e-04)	0.9799 (0.9798 - 0.9801)	0.9786 (0.9785 - 0.9788)	1.0014 (1.0013 - 1.0014)	0.0013 (0.0013 - 0.0014)

© 2023 Jones BE et al. *JAMA Network Open.*

ICU: Yes	634 773	0.0486 (0.048 - 0.0492)	0.0512 (0.0507 - 0.0518)	0.9488 (0.9461 - 0.9512)	-0.0026 (- 0.0028 - - 0.0025)	0.9247 (0.924 - 0.9253)	0.9204 (0.9197 - 0.921)	1.0046 (1.0045 - 1.0048)	0.0043 (0.0041 - 0.0044)
Elixhauser quarrtile: Q1	113 210 8	0.0024 (0.0023 - 0.0025)	0.0025 (0.0024 - 0.0026)	0.9572 (0.9504 - 0.965)	-1e-04 (-1e-04 - -1e-04)	0.9923 (0.9922 - 0.9925)	0.9921 (0.992 - 0.9923)	1.0002 (1.0002 - 1.0002)	2e-04 (2e-04 - 2e-04)
Elixhauser quarrtile: Q2	111 363 2	0.0075 (0.0074 - 0.0077)	0.008 (0.0079 - 0.0082)	0.9409 (0.936 - 0.9464)	-5e-04 (-5e-04 - -4e-04)	0.9847 (0.9845 - 0.9849)	0.984 (0.9837 - 0.9842)	1.0008 (1.0007 - 1.0008)	8e-04 (7e-04 - 8e-04)
Elixhauser quarrtile: Q3	845 756	0.0174 (0.0171 - 0.0177)	0.0186 (0.0183 - 0.0189)	0.9373 (0.933 - 0.9416)	-0.0012 (- 0.0012 - - 0.0011)	0.9704 (0.97 - 0.9708)	0.9685 (0.9681 - 0.9689)	1.0019 (1.0018 - 1.002)	0.0019 (0.0018 - 0.002)
Elixhauser quarrtile: Q4	947 478	0.0435 (0.0432 - 0.044)	0.0468 (0.0465 - 0.0473)	0.9298 (0.9268 - 0.9321)	-0.0033 (- 0.0034 - - 0.0032)	0.9308 (0.9303 - 0.9312)	0.926 (0.9254 - 0.9264)	1.0052 (1.0051 - 1.0054)	0.0048 (0.0047 - 0.005)
Bedsize: 1-99	738 778	0.0161 (0.0158 - 0.0164)	0.0168 (0.0165 - 0.017)	0.9584 (0.9548 - 0.9621)	-7e-04 (-8e-04 - -6e-04)	0.9752 (0.9749 - 0.9756)	0.9741 (0.9738 - 0.9745)	1.0011 (1.001 - 1.0012)	0.0011 (0.001 - 0.0011)
Bedsize: 100-199	147 828 9	0.0165 (0.0163 - 0.0167)	0.0176 (0.0173 - 0.0178)	0.9392 (0.9361 - 0.9421)	-0.0011 (- 0.0011 - - 0.001)	0.97 (0.9698 - 0.9704)	0.9683 (0.9681 - 0.9687)	1.0017 (1.0017 - 1.0018)	0.0017 (0.0016 - 0.0018)
Bedsize: 199-299	803 522	0.0165 (0.0162 - 0.0168)	0.0178 (0.0175 - 0.0181)	0.9266 (0.9223 - 0.931)	-0.0013 (- 0.0014 - - 0.0012)	0.9714 (0.9712 - 0.9719)	0.9694 (0.9692 - 0.9699)	1.002 (1.0019 - 1.0021)	0.002 (0.0019 - 0.0021)
Region: Midwest	630 438	0.0157 (0.0154 - 0.016)	0.0166 (0.0163 - 0.0169)	0.9459 (0.9407 - 0.9505)	-9e-04 (-0.001 - -8e-04)	0.9754 (0.975 - 0.9757)	0.974 (0.9735 - 0.9743)	1.0014 (1.0013 - 1.0015)	0.0014 (0.0013 - 0.0015)
Region: Northeast	308 521	0.0176 (0.0171 - 0.018)	0.0185 (0.0181 - 0.019)	0.9482 (0.9424 - 0.9539)	-0.001 (- 0.0011 - -8e-04)	0.9679 (0.9672 - 0.9685)	0.9662 (0.9656 - 0.967)	1.0017 (1.0015 - 1.0018)	0.0016 (0.0015 - 0.0018)
Region: South	236 952 1	0.0164 (0.0162 - 0.0165)	0.0177 (0.0175 - 0.0178)	0.927 (0.9242 - 0.9294)	-0.0013 (- 0.0013 - - 0.0012)	0.9718 (0.9717 - 0.9721)	0.9699 (0.9697 - 0.9702)	1.002 (1.0019 - 1.002)	0.0019 (0.0019 - 0.002)
Region: West	689 748	0.016 (0.0158 - 0.0164)	0.0172 (0.0169 - 0.0175)	0.9313 (0.9261 - 0.9362)	-0.0012 (- 0.0013 - - 0.0011)	0.9688 (0.9684 - 0.9692)	0.967 (0.9666 - 0.9674)	1.0019 (1.0018 - 1.0021)	0.0019 (0.0018 - 0.002)

© 2023 Jones BE et al. *JAMA Network Open.*

Teaching: No	956499	0.0151 (0.0149 - 0.0153)	0.0163 (0.0161 - 0.0166)	0.9246 (0.9201 - 0.929)	-0.0012 (-0.0013 - -0.0012)	0.9757 (0.9754 - 0.976)	0.9739 (0.9735 - 0.9742)	1.0018 (1.0018 - 1.0019)	0.0018 (0.0017 - 0.0019)
Teaching: Yes	3082475	0.017 (0.0169 - 0.0172)	0.0182 (0.0181 - 0.0184)	0.935 (0.9328 - 0.937)	-0.0012 (-0.0012 - -0.0011)	0.9698 (0.9697 - 0.9701)	0.968 (0.9679 - 0.9683)	1.0019 (1.0018 - 1.0019)	0.0018 (0.0018 - 0.0019)

© 2023 Jones BE et al. *JAMA Network Open.*
