# Supplementary material for: Incidence and Outcomes of Non–Ventilator-Associated Hospital-Acquired Pneumonia in 284 US Hospitals Using Electronic Surveillance Criteria
Source: JAMA Netw Open. 2023 May 18;6(5):e2314185. doi: 10.1001/jamanetworkopen.2023.14185 (PMC10196873; doi:10.1001/jamanetworkopen.2023.14185)
Supplement: Supplement 2. — Data Sharing Statement [file jamanetwopen-e2314185-s002.pdf]

Data Sharing Statement

Jones. Incidence and Outcomes of Non–Ventilator-Associated Hospital-Acquired Pneumonia in 284 US Hospitals Using Electronic Surveillance Criteria. *JAMA Netw Open*. Published May 18, 2023. doi:10.1001/jamanetworkopen.2023.14185

Data
Data available: No

Additional Information
Explanation for why data not available: Due to data security regulations, we are unable to provide datasets, even if they are de-identified. However, we do provide descriptions of data structure and all statistical code through GitHub.
